# LiteCrackSeg: A lightweight hybrid CNN–transformer for efficient crack segmentation

**DOI:** 10.1371/journal.pone.0347765

**Published:** 2026-04-30

**Authors:** Kaleb Amsalu Gobena, MD Youshuf Khan Rakib, Fiseha Berhanu Tesema, Galana Fekadu Asafa, Shengbing Ren

**Affiliations:** 1 School of Computer Science and Engineering, Central South University, Changsha, Hunan, China; 2 School of Computer Science, University of Nottingham Ningbo China, Ningbo, Zhejiang, China; Hohai University, CHINA

## Abstract

Infrastructure cracks are critical indicators of structural deterioration in pavements, bridges, and buildings. Automated crack segmentation has therefore become an important component of structural health monitoring systems. However, accurate pixel-level crack segmentation on resource-constrained devices remains challenging due to the thin, low-contrast, and curvilinear morphology of cracks, as well as severe foreground–background class imbalance. To address these challenges, we propose LiteCrackSeg, a lightweight hybrid CNN–transformer architecture designed for efficient and accurate crack segmentation. The proposed framework adopts a hybrid MobileViT encoder that captures both local spatial details and long-range contextual dependencies while maintaining a compact model size. To enhance morphological sensitivity to elongated crack structures, we introduce a Morphology-Aware MobileViT (MAM-ViT) bottleneck, which integrates dual-branch Dynamic Snake Convolutions (DSConv) to align receptive fields with crack trajectories. Furthermore, a transformer-based decoder with local self-attention progressively reconstructs spatial details, while an attention-guided multi-scale fusion strategy improves boundary precision and structural continuity. To mitigate severe class imbalance, the model is trained using the Tversky loss, which explicitly balances false positives and false negatives. Extensive experiments on three public crack segmentation datasets (DeepCrack, CrackMap, and TUT) demonstrate that LiteCrackSeg achieves state-of-the-art segmentation performance while maintaining high computational efficiency. The proposed model requires only 2.72M parameters and 3.23 GFLOPs, achieving real-time inference at 56 FPS on 512 × 512 images, making it suitable for deployment on resource-constrained edge devices for practical infrastructure inspection.

## Introduction

Cracks are frequently observed defects in pavements, bridges, and buildings; monitoring the quality of these structures is crucial to prevent safety risks [1]. For instance, cracks on road surfaces can quickly develop into large potholes, which can be hazardous for high-speed vehicles, while cracks in bridges can lead to structural collapse, causing significant casualties [2]. These risks highlight the importance of timely and systematic inspection to identify early signs of deterioration. Yet manual visual inspection is time-consuming, subjective, and often infeasible for large-scale infrastructure. This motivates the need for automated structural health monitoring systems that can operate routinely and reliably to prevent catastrophic failures.

In practical deployments, inspection systems increasingly run on resource-constrained edge platforms such as vehicle-mounted cameras, and UAVs equipped with embedded modules like the NVIDIA Jetson series. Streaming high-resolution imagery to remote servers is often infeasible because of bandwidth and latency constraints, and field conditions impose strict power and thermal budgets. As a result, models must deliver accurate, real-time segmentation with small compute and memory footprints suitable for on-device inference.

Early automated approaches relied on traditional image processing for crack extraction [3–5]. These techniques were simple to deploy but degrade under lighting variation, occlusions, and cluttered textures. Convolutional neural networks (CNNs) based methods improve robustness relative to classical pipelines by learning hierarchical local features that capture fine edges and textures, yielding better performance under moderate illumination and background variation [6,7]. However, two limitations remain. First, limited receptive fields hinder modeling of long-range continuity, so predictions can fragment along extended cracks. Second, severe foreground–background imbalance biases learning toward the background, causing missed thin crack pixels unless addressed with imbalance-aware objectives [8].

Vision transformers (ViTs) [9] mitigate the limited-receptive-field issue by modeling an image as patch tokens and using global self-attention to relate distant regions. This global context can help preserve crack continuity and topology under shadows, occlusions, and cluttered backgrounds, and has shown promise on crack imagery [10,11]. However, standard ViT architectures typically have larger model sizes and higher compute, which conflicts with real-time inspection on resource-constrained devices [12].

The hybrid strategy of combining CNNs and transformers harnesses the advantages of CNNs to build a strong, spatially-local feature foundation, and then employs transformers to model global context on top of these rich features. This synergistic combination has yielded strong results for crack segmentation [13–15]. Despite these gains, limitations remain. Many hybrid designs are not explicitly tailored to the slender, curvilinear structure of cracks, limiting the effectiveness of feature fusion. For instance, models that use simple channel-wise concatenation of local patterns and long-range dependencies merely stack representations without enabling deeper interaction. This underuses the complementarity between branches, leading to poor segmentation of fine cracks and a greater susceptibility to background noise [16,17]. Similarly, other models use channel-attention-based fusion [15,18], which can help focus on important cues but tends to neglect spatial information and pixel-level details, compromising overall accuracy. Moreover, jointly optimizing convolutional and transformer branches can increase memory and compute, and complicate training dynamics, hindering deployment on resource-constrained hardware. These observations motivate a morphology-aware, low-overhead design suited to edge platforms.

Many crack segmentation networks, in pursuit of accuracy, deepen architectures and widen channels, accumulating large parameter counts and GFLOPs [17–19]. While such designs can enrich features, their computational cost and power draw hinder edge deployment. Compounding the difficulty, cracks are thin and low-contrast within cluttered backgrounds [20], with severe foreground–background imbalance [8]. Addressing these deployment and data challenges together is essential for practical, reliable crack segmentation in the field.

**Fig 1:**
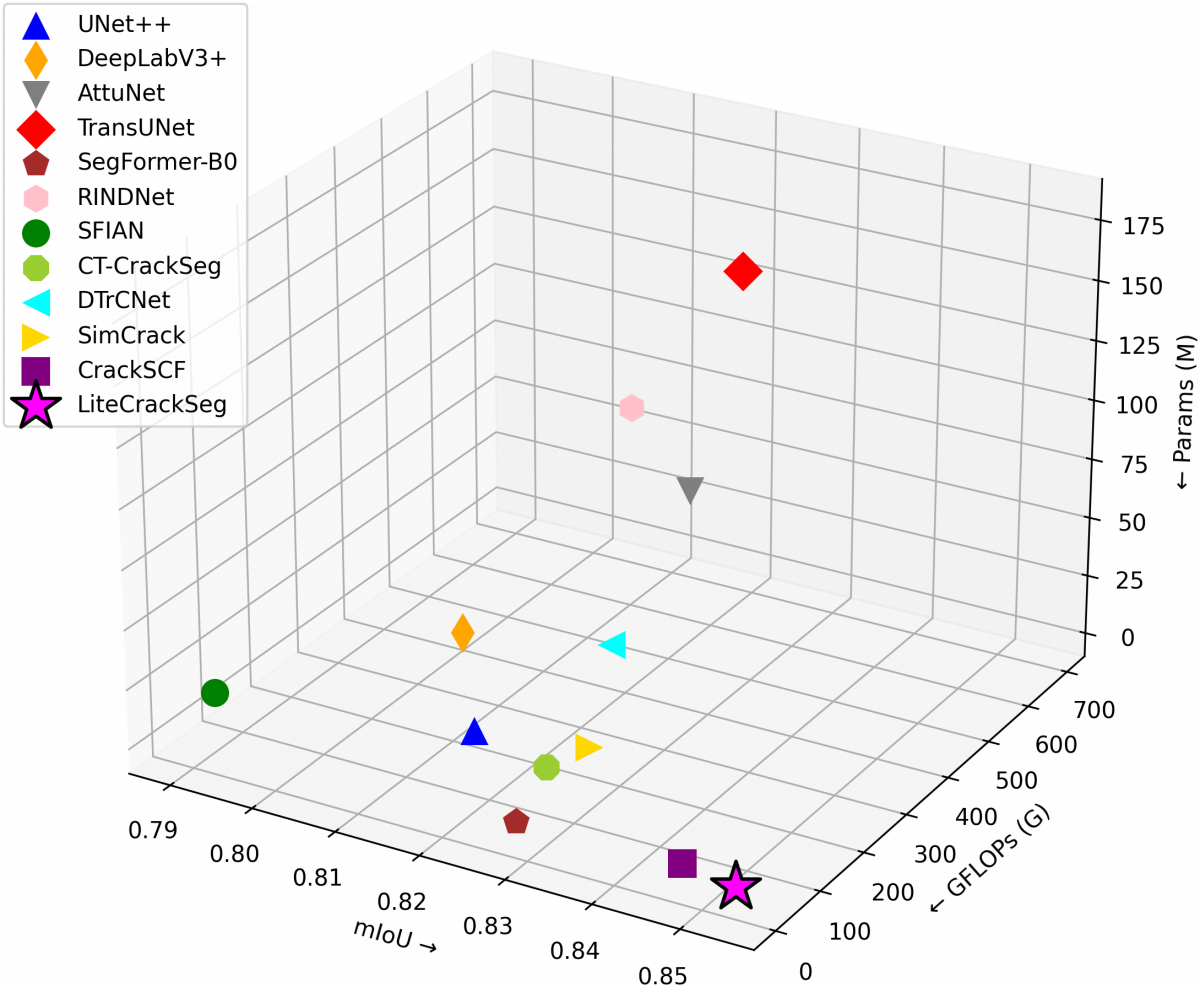
Performance of LiteCrackSeg on TUT dataset compared with SOTA methods.

To meet these demands, we propose LiteCrackSeg, a lightweight hybrid CNN–transformer architecture that produces high quality crack segmentation masks with low computational footprint. LiteCrackSeg incorporates a pre-trained MobileViT [12] encoder branch, which effectively captures both local features through its convolutional components and long-range dependencies via its transformer blocks. To refine these features and suppress irrelevant background cues, lightweight channel attention modules are applied after each encoder block. To explicitly address crack morphology, we introduce a Morphology-Aware MobileViT (MAM-ViT) bottleneck at the deepest semantic stage. This bottleneck leverages dual-branch Dynamic Snake Convolutions (DSConv) [21] to align sampling along crack centerlines, sharpening boundaries and improving continuity in low-contrast regions. For the decoder, we adopt a simplified transformer refinement block inspired by CrackFormer-II [22] to progressively reconstruct spatial detail while controlling computational overhead. The complementary features from the encoder and decoder branches are fused to generate the final segmentation map. Finally, to counter severe class imbalance, we adopt a Tversky-based training objective that places a greater penalty on false positives than on false negatives. This biases the optimizer toward precision, reducing over-segmentation in cluttered scenes. To evaluate our model’s performance and generalization capability, we conduct experiments on three public datasets, namely DeepCrack [23], CrackMap [24], and TUT [25], encompassing challenging conditions like occlusions, complex textures, and uneven illumination. Fig 1 compares the proposed model with SOTA segmentation methods on the TUT dataset, illustrating the performance-complexity trade-off.

The main contributions of this work are summarised as follows:

First, we propose LiteCrackSeg, a unified lightweight transformer-based framework specifically designed for crack segmentation. Unlike prior works that directly adopt heavy transformer encoders or stack multiple refinement blocks, LiteCrackSeg adopts a carefully redesigned architecture that balances morphological sensitivity and computational efficiency.

Second, we introduce a Morphology-Aware MobileViT (MAM-ViT) bottleneck module that integrates dual-branch depthwise separable convolutions with anisotropic receptive fields into a lightweight transformer backbone. This design explicitly enhances the representation of elongated and filamentary crack structures while maintaining low parameter complexity.

Third, we replace conventional global multi-head self-attention (MHSA) with a spatially constrained Local Self-Attention (LSA) mechanism. This redesign reduces computational complexity from $𝒪((HW{)}^{2})$ to $𝒪(HWK_{s}^{2})$ while preserving spatial continuity and improving robustness for thin crack topology reconstruction.

Fourth, we propose an attention-guided multi-scale fusion strategy that dynamically modulates encoder and decoder features at each resolution level via a learnable spatial gate, enabling scale-consistent deep supervision and sharper boundary delineation.

Extensive experiments demonstrate that LiteCrackSeg achieves competitive or superior performance with only 2.72M parameters and 3.23 GFLOPs, validating the effectiveness of the proposed architectural innovations.

## Related work

### Crack segmentation networks

Early research in crack segmentation predominantly relied on CNN-based models to create high-performing crack segmentation networks. Among these, UNet is the most recognized, with its various architectures being widely applied to this task [26–28]. Other notable frameworks used in different studies include FCNs [29,30], DeepLab [31], and SegNet [32]. A classic work in this area is DeepCrack [2], which employs a SegNet-like encoder-decoder structure. Attention mechanisms are frequently paired with CNN-based models to boost performance [33]. For instance, MDAUNet builds on UNet with dual attention modules within an encoder-decoder architecture [27], while another work augmented DeepLabv3+ by adding a multiscale attention mechanism to better aggregate crack patterns [31]. Despite these advances, these methods typically remain constrained by limited receptive fields, often leading to fragmented predictions and susceptibility to background clutter.

Cracks typically have extensive and intricate shapes; therefore, precise segmentation requires capturing both local and global features [34]. In this context, transformer-based models have demonstrated impressive results in crack segmentation. This has led to their increasing use in crack segmentation networks [14,22,35]. Although these methods are very effective at capturing crack textures and reducing background noise, their self-attention mechanism is computationally expensive, with complexity that increases quadratically with the input sequence length. This leads to a high parameter count and significant computational requirements, restricting their deployment on resource-constrained devices.

Recently, transformer-based architectures have also been explored for infrastructure defect detection and crack localisation. For instance, HCTNet introduces a hierarchical cross-transformer framework that improves road crack localisation by enabling multi-scale contextual interaction between features [36]. Similarly, a spatial attention-based dual-stream transformer network has been proposed for concrete defect identification, where spatial attention mechanisms are integrated with transformer encoders to enhance defect feature representation [37]. These studies demonstrate the effectiveness of transformer-based modelling for structural defect analysis. However, many of these transformer-based approaches rely on computationally intensive attention operations or focus primarily on general defect detection rather than fine crack segmentation. In contrast, our proposed LiteCrackSeg architecture is designed as a lightweight hybrid framework that preserves thin crack structures while maintaining computational efficiency suitable for resource-constrained deployment.

More recently, hybrid models that merge CNNs and transformers have gained considerable attention [17,38,39]. The transformer architecture possesses strong long-range modeling capabilities that CNNs lack. Yet, because cracks often constitute a very small portion of an image, a model relying solely on a transformer may be prone to interference from the background, potentially degrading segmentation performance. A hybrid model can effectively mitigate this weakness [15,40]. Since cracks have fine, tubular topological structures, improving the perception to these shapes is key. Qi et al. [21] propose Dynamic Snake Convolution (DSConv) to enhance the geometric sensing of thin, curved structures, leading to significant segmentation gains. However, DSConv is limited by its fixed offset kernel size and a unidirectional learnable offset iteration, which can cause inaccurate iterations and reduce the model’s adaptive capabilities in complex scenes. Building on this, Yu et al. [41] proposed DSCformer model, which enhances DSConv by employing a pyramid kernel and a bi-directional learnable offset update, improving detection of crack structures. However, these networks have drawbacks. They often rely on a single feature fusion method, which can limit their ability to capture both long-range context and local details, which can cause important information in complex areas to be lost. Moreover, their methods prioritize performance while ignoring the significant computational cost of feature extraction and fusion, which makes deployment on resource-constrained devices challenging.

### Addressing class imbalance with loss function

Beyond architectural design, the choice of a loss function is critical, especially for a task like crack segmentation, which exhibits severe foreground–background imbalance. Losses for this task can be broadly categorized into distribution-based, region-based, and compound approaches. Distribution-based losses evaluate per-pixel correctness. Binary Cross-Entropy (BCE) loss [42] is commonly used but can struggle when background pixels vastly outnumber crack pixels. To counteract this imbalance, Weighted Cross-Entropy (WCE) [43] assigns a higher penalty to the minority class. Focal loss [44] down-weights easy, well-classified examples and emphasizes hard crack pixels, helping to mitigate this imbalance. While effective, these methods still treat pixels independently, which can be suboptimal for segmenting thin, continuous structures.

Region-based losses address class imbalance by maximizing the overlap between prediction and ground truth. Among them, Dice loss [45] optimizes regional overlap and is widely used, but it can be unstable for very small or thin structures and, importantly, treats false positives (FP) and false negatives (FN) symmetrically. In practice, the relative cost of FP versus FN is task-dependent. Tversky loss [46] generalizes Dice by introducing asymmetric FP/FN weights, allowing the precision–recall balance to be adjusted.

More recent works have explored compound losses that combine distribution-based and region-based objectives, such as BCE-Dice hybrid loss [25]. While these hybrid losses can be powerful, they introduce additional complexity and hyperparameters. In our work, we contend that for crack segmentation, the key advantage is the ability to tune the precision–recall balance via asymmetric FP/FN penalties. We therefore adopt the Tversky loss [46] as our primary training objective.

### Lightweight networks

Deploying deep learning models on resource-constrained devices requires architectures that operate efficiently under limited computational and memory resources. This need has motivated the development of compact, high-performance networks for real-time, low-latency applications. Foundational architectures, such as MobileNet [47] and ShuffleNet [48], pioneered depthwise separable and channel-shuffling operations, respectively, to substantially reduce computation and parameter count while maintaining strong accuracy. Building on these convolutional innovations, MobileViT [12] extends efficiency to transformer-based architectures by integrating convolutional locality with transformer-based global reasoning, offering a powerful, lightweight solution for mobile vision tasks.

Recently, researchers have started integrating these techniques into crack segmentation models for real-time performance on resource-constrained devices. RHACrackNet [49], introduced a lightweight network that incorporates residual blocks and hybrid attention mechanisms for real-time crack detection. Hui et al. [25] proposed CrackSCF model, which effectively reduces the computational load and parameter count during training and inference by replacing all convolution operations with a method that fuses local patterns and pixel dependencies. EfficientCrackNet [50] further exemplifies this trend toward efficiency by designing a compact hybrid encoder–decoder with depthwise separable convolutions and MobileViT blocks for joint local–global reasoning. Despite reducing computational demand, these lightweight models often sacrifice model expressiveness by focusing too heavily on parameter reduction. This prevents them from effectively capturing complex features, leading to poor segmentation results.

Our work addresses these gaps by developing LiteCrackSeg, a lightweight hybrid framework designed to enhance segmentation efficiency without compromising performance. We argue that the primary limitation of current hybrid models is not fusion but inefficient feature extraction under tight computational constraints. We adopt a pre-trained MobileViT backbone, leveraging its hybrid convolutional-transformer design to capture both local and global features while maintaining the low parameter count. To overcome the limitations of standard convolutions, we introduce a novel Morphology-Aware MobileViT (MAM-ViT) bottleneck. This module extends the core concept of DSConv; although DSConv can be limited by its unidirectional iteration, our dual-branch module (DSConv-*x*, DSConv-*y*) explicitly traces curvilinear paths in dual directions, enhancing robustness for complex crack geometries. To further ensure accurate crack segmentation despite the severe class imbalance, we employ the Tversky loss during training, which allows us to set the FP-FN weighting, a critical consideration since missing cracks is far riskier than slight over-segmentation. The final segmentation mask is produced through progressive, attention-guided feature fusion and upsampling, achieving SOTA accuracy without the computational burden of larger models.

## Methodology

**Fig 2:**
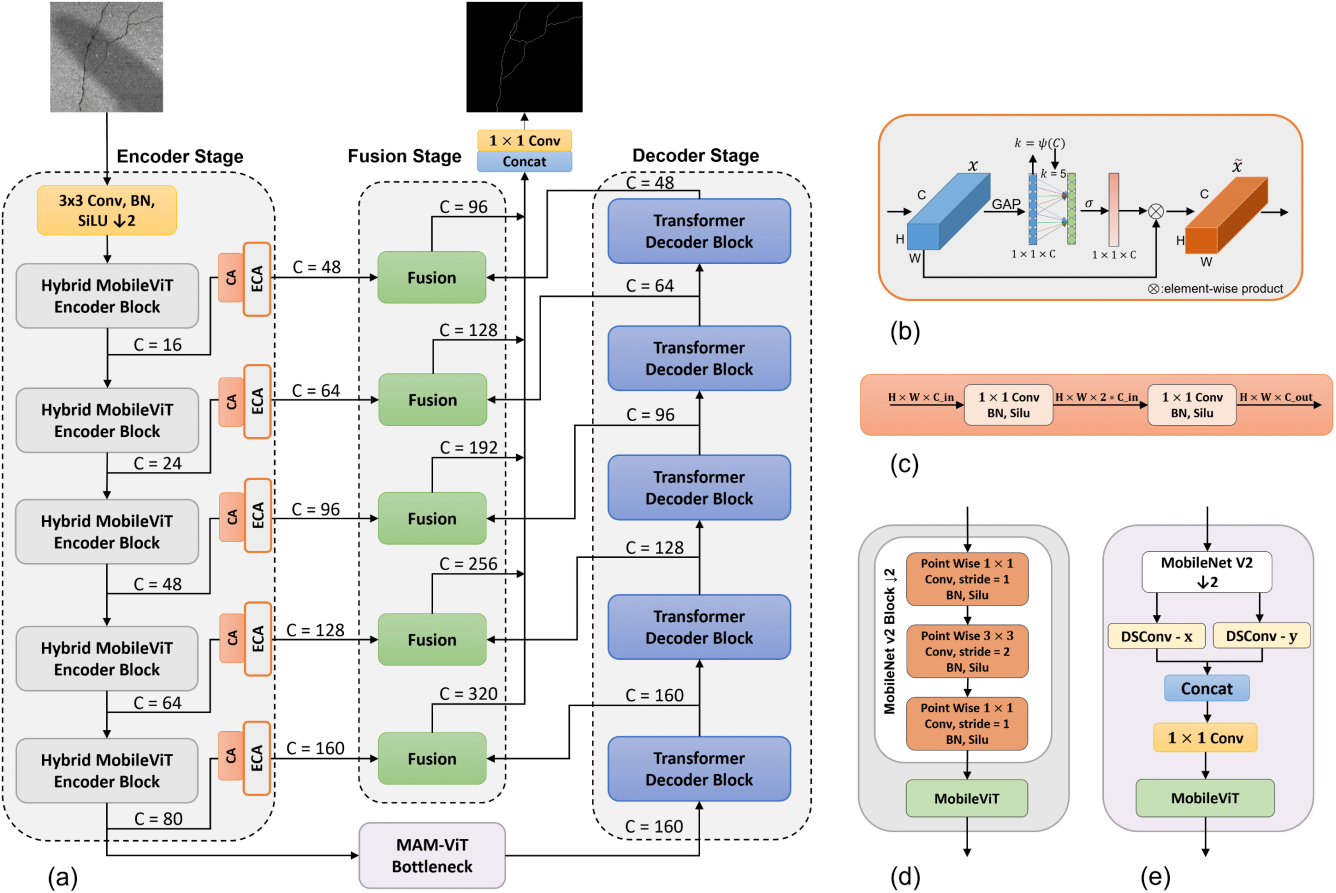
LiteCrackSeg Architecture. **(a)** Main LiteCrackSeg architecture illustrating a hybrid encoder–decoder that extracts and fuses coarse and fine-grained features end-to-end. **(b)** ECA (Efficient Channel Attention) module, which adaptively reweights channel features to emphasize informative crack signals. **(c)** CA (Channel Adapter) module, which aligns pretrained MobileViT channels to target dimensions via lightweight expansion-projection for efficient feature adaptation. **(d)** Hybrid MobileViT encoder block for multi-scale local-global representation learning. **(e)** Morphology-aware MobileViT (MAM-ViT) bottleneck that first leverages dual-branch Dynamic Snake Convolution (DSConv) to model tubular structures, followed by a MobileViT block to re-integrate long-range context efficiently.

The overall architecture of LiteCrackSeg is illustrated in Fig 2(a). It is a lightweight encoder-decoder framework designed for end-to-end pixel-level segmentation. The network’s encoder consists of a lightweight pre-trained MobileViT backbone that efficiently extracts hybrid local-global features, which are further refined by channel attention mechanisms. At the network’s deepest layer, a novel Morphology-Aware MobileViT (MAM-ViT) bottleneck is introduced, leveraging dual-branch Dynamic Snake Convolutions (DSConv) to capture the unique geometry of cracks. Following the bottleneck, a transformer-based decoder progressively reconstructs high-resolution feature maps.

This five-scale hierarchical design is crucial for crack segmentation as it refines features at multiple scales. The high-resolution stages capture fine-grained local details like thin crack edges, while the low-resolution stages develop a larger receptive field to understand the global context and long-range connectivity of the crack network. Our proposed five stages provide a sufficient depth to balance robust feature extraction with our lightweight objective. To produce the final prediction, an attention-guided multi-scale fusion head combines encoder and decoder features at each scale to produce side maps. These maps are upsampled, concatenated, and compressed with a final 1 × 1 convolution. Our primary contributions are (1) a hybrid local–global encoder redesigned for morphological sensitivity via channel-attention filtering, and (2) the novel MAM–ViT bottleneck for morphological alignment. While our decoder draws inspiration from the hierarchical refinement philosophy of CrackFormer-II [22], we fundamentally re-engineer these stages to be edge-compatible. Specifically, we replace heavy global attention with a spatially constrained Local Self-Attention (LSA) mechanism and introduce an attention-guided multi-scale fusion strategy. This ensures high-resolution refinement for thin structures while achieving a significantly lower computational footprint suitable for edge deployment.

### Hybrid MobileViT encoder

By leveraging the complementary strengths of CNNs and transformers, our encoder maximizes representational power while substantially minimizing computational burden and parameter count. The feature extraction process can be conceptually divided into two phases: first, CNN-based local feature extraction and downsampling, and second, MobileViT-based local–global representation learning.

In the first phase, the early layers of the encoder adopt MobileNetV2-style convolutions to extract low-level features while progressively reducing spatial resolution. As illustrated in Fig 2(d), which details the structure of a single hybrid MobileViT encoder block, first the MobileNetV2 blocks handle efficient local processing. This approach preserves edge-like patterns characteristic of thin, elongated cracks while lowering the spatial resolution, which in turn limits the quadratic cost of self-attention in the subsequent phase. Compared with purely transformer encoders, this initial CNN-based stage injects a strong spatial inductive bias, establishing a reliable foundation for boundary-sensitive crack features.

In the second phase, the downsampled feature map is processed by the MobileViT blocks within each hybrid MobileViT encoder block to capture local detail and global context. Convolutional operations first encode spatial structure and enrich feature expressivity. To capture broader context, the resulting feature map is divided into non-overlapping windows, each window is reshaped into a token sequence, and a lightweight self-attention layer is applied to capture interactions between spatially distant regions. We specifically selected the pre-trained extra-extra-small (XXS) variant as it provides a powerful yet parameter-efficient mechanism for this task, aligning with our objective of creating a lightweight architecture. This dual mechanism preserves pixel-level continuity while enabling the encoder to reason about broader structural context across large surfaces.

To harmonize the outputs from this pre-trained backbone with the decoder’s requirements, each of the five hidden states extracted from the encoder, with original channel dimensions of {16, 24, 48, 64, 80}, is passed through a lightweight Channel Adapter (CA), inspired by inverted bottlenecks [47]. This projection to a higher-dimensional feature space of {48, 64, 96, 128, 160} serves as a critical feature enrichment step. It enhances the representational capacity of the compact encoder features, providing the fusion stage with a richer set of details necessary for segmenting fine, low-contrast cracks. The specific channel dimensions were chosen to strike an effective balance, boosting segmentation performance without compromising the model’s overall lightweight efficiency.

Given an encoder feature $F^{k}\in {\mathbb{R}}^{H_{k}\times W_{k}\times C_{k}}$, the Channel Adapter performs a lightweight projection:


$${\tilde{F}}^{k}=\phi_{2}\;\left(\delta \;\left(\phi_{1}(F^{k})\right)\right),$$ (1)

where $\phi_{1}$ and $\phi_{2}$ denote 1 × 1 convolutions for channel expansion and projection, respectively, and δ(·) denotes the GELU activation function.

Immediately after adaptation, an Efficient Channel Attention (ECA) module [51] injects inter-channel context to adaptively reweight informative crack features. Given the adapted feature ${\tilde{F}}^{k}\in {\mathbb{R}}^{H_{k}\times W_{k}\times C_{k}}$, global average pooling is first applied along the spatial dimensions:


$$z_{c}=\frac{1}{H_{k}W_{k}}\sum_{i=1}^{H_{k}}\sum_{j=1}^{W_{k}}{\tilde{F}}^{k}(i,j,c),$$ (2)

where *c* indexes the channel dimension.

A lightweight 1D convolution ψ(·) is applied to the aggregated channel descriptor vector $𝐳\in {\mathbb{R}}^{C_{k}}$ (formed by concatenating all *z*_*c*_) to capture local cross-channel interactions:


ω=σ(ψ(𝐳)), (3)

where σ(·) denotes the Sigmoid function. The refined skip feature is obtained by channel-wise reweighting:


$$E^{k}=\omega ⊙{\tilde{F}}^{k},$$ (4)

where ⊙ denotes element-wise multiplication, with the channel attention weights ω broadcasted across the spatial dimensions *H*_*k*_ × *W*_*k*_.

This two-step refinement enforces dimensional consistency for multi-scale fusion and selectively enhances informative responses while suppressing irrelevant background cues. For crack segmentation, this projection-plus-attention design is especially beneficial: it mitigates the ImageNet-to-crack domain mismatch, stabilizes optimization dynamics, and amplifies filamentary crack signals against noisy textures such as asphalt aggregates or concrete spalling. The result is a compact yet expressive set of multi-scale features that are locally detailed, globally contextualized, and optimally conditioned for the attention-guided fusion pathway.

### Morphology-aware MobileViT (MAM-ViT) bottleneck

Crack segmentation is challenging because thin, elongated, and curvilinear structures violate the rigid, grid-like receptive fields of standard convolutions. To explicitly encode this morphology, we introduce a MAM-ViT bottleneck at the deepest, most semantic level. The bottleneck begins with a MobileNetV2 downsampling block to control compute, followed by a morphology-aware deformation stage and a MobileViT block that restores long-range context.

At the core of the deformation stage is Dynamic Snake Convolution (DSConv), which learns a two-dimensional offset field that deforms an axis-aligned sampling kernel so the sampling path follows crack centerlines. To be robust to orientation, we employ two parallel branches: a horizontal kernel with vertical deformation (denoted DSConv-*y*) and a vertical kernel with horizontal deformation (DSConv-*x*). The two responses are concatenated and merged with a 1 × 1 projection before entering the MobileViT block.

**Fig 3:**
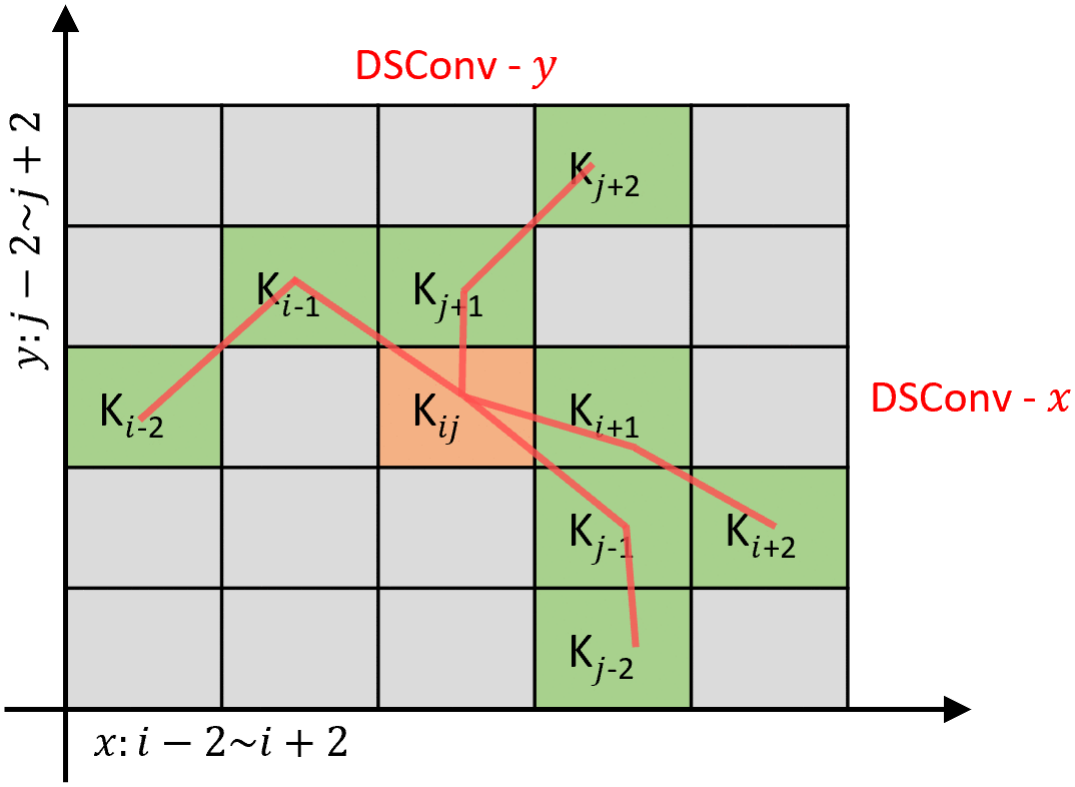
Deformed receptive field of Dynamic Snake Convolution (DSConv).

Offsets are predicted by a lightweight CNN, bounded to [−1, 1] with tanh, and applied via center-anchored cumulative updates along the kernel axis. This keeps the sampling trajectory attached to the target structure and yields smooth, crack-aligned paths. Because the learned offsets are fractional, features are sampled at the deformed coordinates using bilinear interpolation. Let the kernel length be an odd number *K* = 2*s* + 1 with offset index m∈{0,…,s} and center (*x*_*i*_, *y*_*i*_). In this context, $\Delta x_{l}$ and $\Delta y_{l}$ represent the learned fractional offsets at the *l*-th spatial step along the cumulative deformation path, while *K*_*i*±*m*_ and *K*_*j*±*m*_ denote the final deformed coordinates of the sampling points. Fig 3 illustrates these updates.

For the branch with a horizontal kernel and vertical deformation (DSConv-*y*; sampling along *x*, deforming in *y*), is shown in Eq. 5.


$$K_{i\pm m}=\left\{ \begin{array}{c} (x_{i+m},y_{i+m})=(x_{i}+m,\;y_{i}+\sum_{l=i}^{i+m}\Delta y_{l}),\\ (x_{i-m},y_{i-m})=(x_{i}-m,\;y_{i}+\sum_{l=i-m}^{i}\Delta y_{l}), \end{array}\right.$$ (5)

For the branch with a vertical kernel and horizontal deformation (DSConv-*x*; sampling along *y*, deforming in *x*), is shown in Eq. 6.


$$K_{j\pm m}=\left\{ \begin{array}{c} (x_{j+m},y_{j+m})=(x_{j}+\sum_{l=j}^{j+m}\Delta x_{l},\;y_{j}+m),\\ (x_{j-m},y_{j-m})=(x_{j}+\sum_{l=j-m}^{j}\Delta x_{l},\;y_{j}-m). \end{array}\right.$$ (6)

The two deformed responses are then concatenated channel-wise and compressed by a 1 × 1 convolution, yielding a morphology-aligned representation that captures filamentary structure in both axial directions. This entire morphology-aware feature map is subsequently fed into a conventional MobileViT module to re-integrate global context, enabling the network to bridge gaps and enforce shape consistency before decoding.

### Transformer decoder with attention

Unlike CrackFormer-II [22], which employs stacked transformer refinement blocks with global attention, our design adopts a single lightweight transformer block per stage combined with local self-attention. This modification is not merely a simplification but a principled redesign aimed at improving computational efficiency while maintaining spatial refinement capability for thin crack structures.

**Fig 4:**
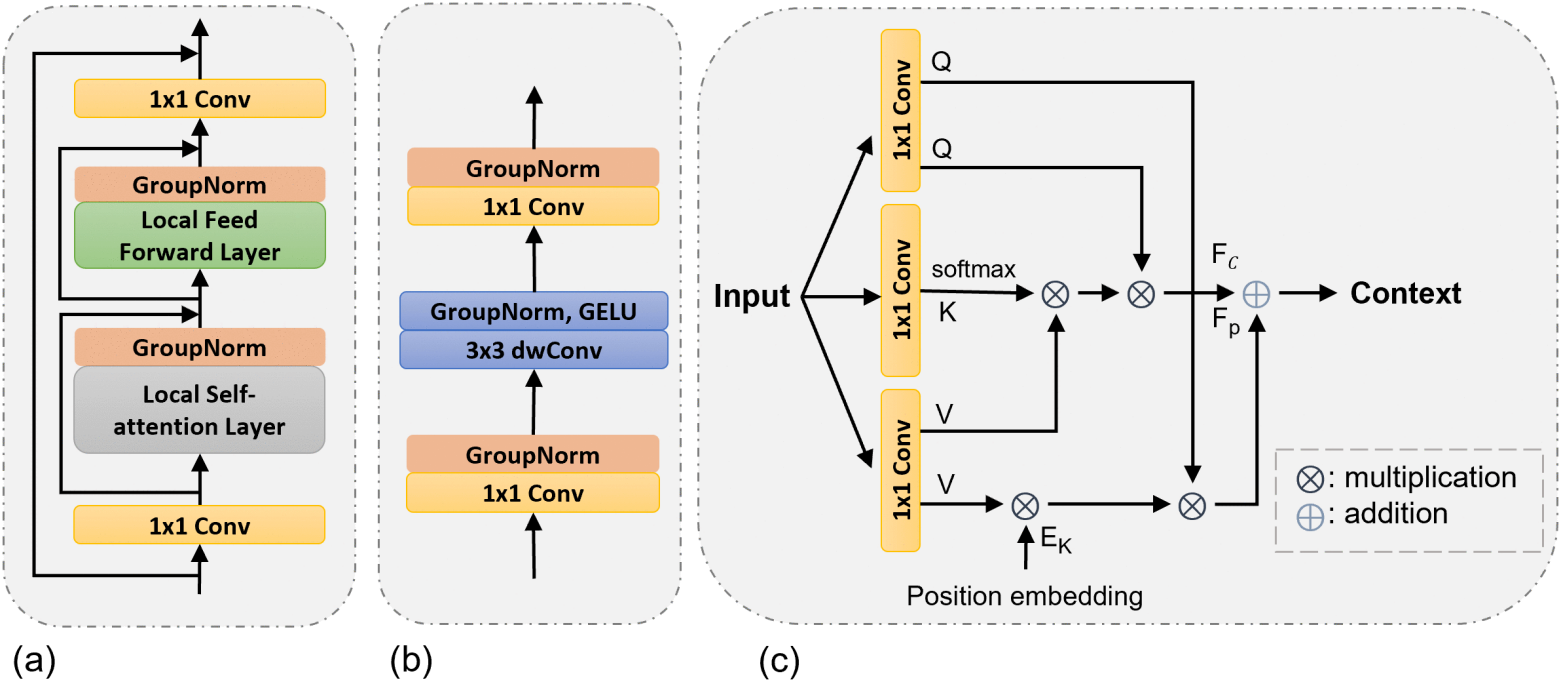
(a) Transformer decoder block (TDB) to dynamically adapt features during the decoder stage. (b) Local Feed-Forward layer. (c) Local self-attention layer.

After deep feature encoding and the morphology-aware bottleneck, the decoder reconstructs spatial detail through five sequential upsampling stages. At each stage of the decoder, the incoming feature map is enlarged a factor of 2 using bilinear interpolation, then refined by a bottlenecked transformer decoder block (TDB), as shown in Fig 4(a). A 1 × 1 projection first reduces the channels by a ratio *r* = 4; the core transformer sub-block mixes information; and a second 1 × 1 projection restores the target width. A residual shortcut spanning the block stabilizes optimization and preserves low-frequency content.

The Local Self-Attention (LSA) layer (Fig 4(c)) retains the 2D tensor layout.

Given an input feature map $X\in {\mathbb{R}}^{H\times W\times C}$, queries, keys, and values are computed as:


$$Q=W_{Q}X,\;K=W_{K}X,\;V=W_{V}X,$$ (7)

where *W*_*Q*_, *W*_*K*_, and *W*_*V*_ are learnable 1 × 1 convolution projections and *d* is the channel dimension. In practice, the LSA module uses *h* = 4 attention heads. For a feature dimension *C*, each head operates on a subspace of dimension *d* = *C*/*h*. Let $Q_{i},K_{i},V_{i}\in {\mathbb{R}}^{d}$ represent the specific query, key, and value vectors extracted at spatial index *i*.

Local attention is computed within a sliding window Ω(i) of size *K*_*s*_ × *K*_*s*_ centered at spatial index *i*:


$$Attn(X_{i})=\sum_{j\in \Omega (i)}Softmax\;\left(\frac{Q_{i}^{⊤}K_{j}}{\sqrt{d}}\right)V_{j}.$$ (8)

In addition to the content-based attention term defined above, a learnable relative-position kernel is convolved with *V* within the same local window Ω(i) to encode spatial priors. The final attention response is obtained by summing the content and positional components:


$${\tilde{X}}_{i}=Attn(X_{i})+Pos(X_{i}),$$ (9)

where Pos(·) denotes the relative-position response.

A residual connection then produces the output:


$$X_{i}^{\prime }=X_{i}+{\tilde{X}}_{i}.$$ (10)

The computational complexity of global self-attention scales as $𝒪((HW{)}^{2})$, whereas local attention reduces this to $𝒪(HWK_{s}^{2})$, which is substantially lower when $K_{s}\ll HW$. Since the encoder and convolutional operations scale linearly with spatial resolution, the overall complexity of LiteCrackSeg remains approximately $𝒪(HWK_{s}^{2})$, thereby avoiding the quadratic scaling behaviour associated with global self-attention.

Replacing global MHSA with LSA therefore significantly reduces computational cost while maintaining segmentation accuracy.

Compared with conventional multi-head self-attention (MHSA), which models long-range dependencies across the entire spatial domain, the proposed local self-attention (LSA) restricts interaction to spatial neighborhoods. This locality constraint is particularly beneficial for crack segmentation, where structural continuity and boundary precision are more critical than global semantic context. By focusing on local spatial coherence, LSA enhances thin-structure preservation, reduces noise propagation, and improves robustness without incurring unnecessary quadratic overhead.

The Local Feed-Forward (LFF) layer (Fig 4(b)) is a spatially aware MLP.

Formally, the LFF transformation is expressed as:


$$LFF(X)=W_{2}\;\delta \;\left({DWConv}_{3\times 3}\left(W_{1}X\right)\right),$$ (11)

where *W*_1_ and *W*_2_ are 1 × 1 convolution projections, DWConv denotes depthwise convolution, and δ(·) represents the GELU activation.

By incorporating a depthwise convolution, the LFF layer enhances local feature aggregation in a parameter-efficient manner, which is crucial for reconstructing fine crack textures without the computational cost of standard MLP layers. LFF improves locality and optimization compared with a purely point-wise MLP. Residual connections wrap both LSA and LFF before the outer bottleneck skip is applied. Repeating the upsampling followed by transformer refinement across five scales progressively restores high-frequency detail and sharpens boundaries, yielding representations that are well prepared for the fusion stage.

### Attention-guided multi-scale fusion

To produce a crisp, topology-faithful crack mask, we used multi-scale attention-guided fusion in parallel with the decoder. At each resolution level *k*, the refined encoder feature *E*^*k*^ and the decoder feature *D*^*k*^ at the same scale are first aligned in spatial size. A context-aware spatial gate is then computed by summing the aligned tensors, passing the result through a lightweight convolutional stack, and applying a Sigmoid, as shown in Eq. 12.


$$\gamma^{k}=\sigma \;\left({Conv}_{3\times 3}\;\left(GN\;\left({Conv}_{3\times 3}\;\left(GN\;\left(GELU\;(E^{k}+D^{k})\right)\right)\right)\right)\right).$$ (12)

Where σ denotes the Sigmoid activation function, GELU is the nonlinearity and GN denotes GroupNorm.

Next, the encoder and decoder features are fused by a separable bottleneck and modulated by the gate to produce a per-scale side prediction; the side map is then bilinearly upsampled to the input resolution, as shown in Eq. 13.


$$S_{\text{side}}^{k}=Up\;\left({Conv}_{1\times 1}\;\left(\gamma^{k}⊙\phi ([E^{k},\;D^{k}])\right)\right).$$ (13)

Where [·,·] is channel concatenation, ⊙ is element-wise multiplication, ϕ(·) is a depthwise/grouped 3 × 3 followed by a pointwise 1 × 1 with SiLU, and Up(·) denotes bilinear upsampling to the original input resolution.

Finally, the five side outputs $\{S_{\text{side}}^{k}{\}}_{k=1}^{5}$ are concatenated along the channel dimension and are fused by a final ${Conv}_{1\times 1}$ to produce the final single-channel prediction map. This deep-supervised, scale-consistent fusion leverages encoder detail and decoder context at every level, yielding sharp boundaries and continuous, filamentary crack maps.

### Loss function for imbalance

Crack segmentation is a classic class-imbalanced problem where background pixels vastly outnumber crack pixels. To address this, specialized loss functions can re-balance the training objective by giving more weight to the minority class. To explicitly adjust the balance between FP and FN, we adopt the Tversky loss [46], whose index is given in Eq. 14. Unlike the symmetric Dice loss, Tversky introduces coefficients α and β to differentially weight FP and FN:


$$TI(\alpha ,\beta )=\frac{TP+\varepsilon }{TP+\alpha \;FP+\beta \;FN+\varepsilon },\;\beta =1-\alpha ,\;\varepsilon =10^{-5}.$$ (14)

Given binary logits *v* and labels *y*, probabilities p=σ(v) are used to compute TP, FP, and FN over all pixels. Specifically, for *P* total pixels, these are computed as: $TP=\sum_{n=1}^{P}p_{n}y_{n}$, $FP=\sum_{n=1}^{P}p_{n}(1-y_{n})$, and $FN=\sum_{n=1}^{P}(1-p_{n})y_{n}$. The loss is defined in Eq. 15:


$$L_{\text{Tversky}}=1-TI(\alpha ,\beta ).$$ (15)

In our experiments, we set α=0.75 (thus β=0.25). This setting asymmetrically weights false positives and false negatives to find an optimal balance for this task. We selected this value as it yielded the best-performing model on our validation set, providing a robust balance between capturing fine cracks and avoiding background noise.

Algorithm 1: LiteCrackSeg training and inference

**Input:** Training set $𝒟=\{(I_{i},Y_{i}){\}}_{i=1}^{N}$, epochs *T*, learning rate η, Tversky parameters (α,β), threshold τ.

**Output:** Trained parameters $\theta^{*}$ and predicted mask $\hat{Y}$ for a test image *I*.

**Training:**

1. Initialise parameters $\theta =\{\theta_{enc},\theta_{CA},\theta_{ECA},\theta_{MAM},\theta_{dec},\theta_{fuse}\}$.

2. For each epoch t=1,…,T:

(a) Sample a minibatch (*I*,*Y*) from 𝒟.

(b) Encoder: $\left\{F^{k}{\}}_{k=1}^{5}\leftarrow MobileViTEnc(I;\theta_{enc}\right)$.

(c) Skip refinement: $E^{k}\leftarrow ECA(CA(F^{k};\theta_{CA});\theta_{ECA})$.

(d) Bottleneck: $B\leftarrow MAM\;-\;ViT(E^{5};\theta_{MAM})$ (dual-branch DSConv + MobileViT).

(e) Decoder: $\left\{D^{k}{\}}_{k=1}^{5}\leftarrow DecoderTDB(B,\{E^{k}\};\theta_{dec}\right)$.

(f) Fusion head: $\hat{Y}\leftarrow Fusion(\{E^{k}\},\{D^{k}\};\theta_{fuse})$.

(g) Compute loss: $L\leftarrow L_{Tversky}(\hat{Y},Y;\alpha ,\beta )$.

(h) Update θ using AdamW and the LR scheduler.

3. Return $\theta^{*}$.

**Inference:** For a test image *I*, compute $\hat{Y}$ using steps b–f and output the binary mask $\tilde{Y}=𝕀[\hat{Y}\geq \tau ]$.

For clarity and reproducibility, the overall architectural configuration of LiteCrackSeg is summarised in Table 1. The encoder consists of five hierarchical stages with output channel dimensions {16, 24, 48, 64, 80}, respectively. Each stage contains a lightweight convolutional block followed by MobileViT-style hybrid processing. The final bottleneck integrates the proposed MAM-ViT module with dual-branch depthwise separable convolutions. The decoder mirrors the encoder with five sequential upsampling stages. Each stage contains a single Transformer Decoder Block (TDB), composed of one Local Self-Attention (LSA) layer followed by one Local Feed-Forward (LFF) layer. The LSA module employs *h* attention heads (set to *h* = 4 in our implementation) with channel dimension *d* = *C*/*h*, where *C* denotes the stage feature width.

**Table 1:** LiteCrackSeg architecture configuration. Feature dimensions are channel sizes. *H* × *W* denotes spatial resolution relative to input.

Stage	Resolution	# Blocks / Layers	Feature dim	# Heads
Input	H×W	–	3	–
**Encoder (MobileViT-XXS) + CA + ECA**				
E1 (early conv)	$\frac{H}{2}\times \frac{W}{2}$	1 Conv	48	–
E2 (MV2)	$\frac{H}{4}\times \frac{W}{4}$	3 MV2 blocks	64	–
E3 (MobileViT)	$\frac{H}{8}\times \frac{W}{8}$	2 Transformer layers	96	4
E4 (MobileViT)	$\frac{H}{16}\times \frac{W}{16}$	4 Transformer layers	128	4
E5 (MobileViT)	$\frac{H}{32}\times \frac{W}{32}$	3 Transformer layers	160	4
**Bottleneck (MAM-ViT)**				
MV2 Downsample	$\frac{H}{64}\times \frac{W}{64}$	1 MV2Block	144	–
Morphology-aware stage	$\frac{H}{64}\times \frac{W}{64}$	DSConv-*x*/*y* + MobileViT (3 layers)	144	4
Channel Projection	$\frac{H}{64}\times \frac{W}{64}$	1 × 1 conv	160	–
**Decoder (5-stage upsampling)**				
D5	$\frac{H}{32}\times \frac{W}{32}$	1 TDB	160	4
D4	$\frac{H}{16}\times \frac{W}{16}$	1 TDB	128	4
D3	$\frac{H}{8}\times \frac{W}{8}$	1 TDB	96	4
D2	$\frac{H}{4}\times \frac{W}{4}$	1 TDB	64	4
D1	$\frac{H}{2}\times \frac{W}{2}$	1 TDB	48	4
**Multi-Scale Fusion**				
Side maps (k=1…5)	*H* × *W*	5 concat(E_*k*_, D_*k*_)	1 each	–
Final prediction	*H* × *W*	1 × 1 conv (5 → 1)	1	–

## Experimental analysis

### Dataset

We conducted experiments on three public crack segmentation datasets: DeepCrack [23], CrackMap [24], and TUT [25]. DeepCrack is a widely used benchmark containing 537 images (544 × 384) of asphalt and concrete cracks captured from diverse viewpoints and under various conditions, including fine, wide, stained, and fuzzy cracks. CrackMap consists of 120 images (256 × 256) focusing on thin and complex bitumen pavement cracks. While both datasets are useful for benchmarking, their relatively simple crack morphologies and limited scenarios may not fully test a network’s generalization. To address this, we also utilized the more challenging TUT dataset, which contains 1408 images (640 × 640) spanning eight distinct scenarios, including bitumen, cement, bricks, and metal surfaces. Its diversity in materials, complex backgrounds, occlusions, and uneven lighting provides a rigorous test for model robustness in real-world conditions.

A key challenge across all crack segmentation datasets is severe class imbalance, where crack pixels represent only a tiny percentage of all pixels, as shown in Table 2. By evaluating our model across datasets that vary in scale, complexity, and crack appearance, we can rigorously assess both its performance and generalization. Importantly, these datasets align with our focus on a lightweight architecture that efficiently handles class imbalance while maintaining high accuracy across diverse real-world scenarios.

**Table 2:** Comparison of crack and non-crack pixels in the DeepCrack, CrackMap, and TUT datasets.

Datasets	Crack pixels (%)	Non-crack pixels (%)
DeepCrack	3.54	96.46
CrackMap	4.10	95.90
TUT	3.16	96.84

### Evaluation metrics

To evaluate the performance of our proposed crack segmentation model, we use six metrics: Precision (P), Recall (R), F1 score, Optimal Dataset Scale (ODS), Optimal Image Scale (OIS), and mean Intersection over Union (mIoU). The ODS indicates the best score obtained on the entire dataset using a fixed threshold *m* that is applied uniformly to the whole dataset, the formula is given in Eq. 16, while OIS indicates the average of the best per-image scores with an ideal threshold *n* chosen for every image individually, the formula is given in Eq. 17.


$$ODS=\max_{m}\frac{2\cdot P_{m}\cdot R_{m}}{P_{m}+R_{m}}$$ (16)

$$OIS=\frac{1}{N}\sum_{a=1}^{N}\max_{n}\frac{2\cdot P_{n,a}\cdot R_{n,a}}{P_{n,a}+R_{n,a}}$$ (17)

Here, *N* denotes the total image count.

The mIoU measures the average overlap between the predicted mask and the ground truth label, the formula is given in Eq. 18.


$$MIoU=\frac{1}{i+1}\sum_{a=0}^{i}\frac{p_{aa}}{\sum_{b=0}^{i}p_{ab}+\sum_{b=0}^{i}p_{ba}-p_{aa}}$$ (18)

where *i* denotes the total number of classes, and *p*_*ab*_ counts the pixels whose ground-truth label is class *a* but are assigned to class *b* the predicted class. For binary segmentation, we set *i* = 1.

Finally, AUC-ROC evaluates diagnostic ability by plotting the True Positive Rate ($TPR=\frac{TP}{TP+FN}$) against the False Positive Rate ($FPR=\frac{FP}{FP+TN}$) across varying decision thresholds. The AUC provides an aggregate measure of performance across all possible classification levels.

### Training dynamics analysis

**Fig 5:**
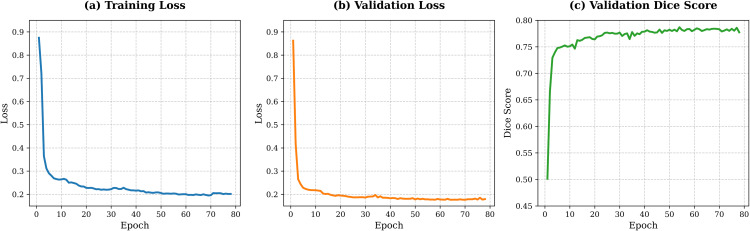
Training dynamics of LiteCrackSeg. **(a)** Training loss, (b) validation loss, and (c) validation Dice score over training epochs. The curves show stable convergence, with validation performance stabilising around epoch 78.

To further illustrate the training stability and convergence behaviour of LiteCrackSeg, we analyse the evolution of training loss, validation loss, and validation Dice score during training. As shown in Fig 5, the training loss decreases rapidly during the early epochs and gradually converges as the model stabilises. The validation loss follows a similar trend, indicating consistent generalisation behaviour without significant overfitting.

The validation Dice score increases steadily and stabilises after approximately epoch 78, suggesting that the model reaches a stable segmentation performance near the end of training. These curves demonstrate that LiteCrackSeg converges smoothly and maintains stable optimisation behaviour throughout the training process.

### Experimental setup

All experiments were conducted using the PyTorch framework on a single NVIDIA GeForce RTX 4090 GPU. In training, we set the initial learning rate to 1 × 10^−4^, and we employ the AdamW optimizer $\left(\beta_{1}=0.9,\;\beta_{2}=0.999\right)$. A cosine-annealing scheduler with warm restarts controls the learning rate $\left(T_{0}=10,\;T_{\text{mult}}=2,\;\eta_{\min}=10^{-6}\right)$. The weight decay is set to 0.01 and the batch size is 4. We trained our models for a total of 100 epochs. We used early stopping with a patience of 25 epochs, and the checkpoint with the highest ODS on the validation set was saved as the best model. To improve generalization, we applied on-the-fly data augmentation to the training set using the Albumentations library, including color jitter, Gaussian blur and noise, image compression, random flips, and minor affine transformations (shift, scale, rotate). Validation did not use augmentation. For clarity and reproducibility, the key training hyperparameters are summarised in Table 3.

**Table 3:** Training and architectural hyperparameters of LiteCrackSeg.

Hyperparameter	Value
Framework	PyTorch
GPU	NVIDIA RTX 4090
Optimizer	AdamW
Initial learning rate	1 × 10^−4^
$\beta_{1},\beta_{2}$	0.9, 0.999
Learning rate scheduler	Cosine annealing with warm restarts
*T*_0_	10
*T*_mult_	2
$\eta_{\min}$	10^−6^
Weight decay	0.01
Batch size	4
Epochs	100
Early stopping patience	25
Model selection metric	Validation ODS
Attention heads (*h*)	4
Local window size (*K*_*s*_)	7
Tversky α	0.75
Tversky β	0.25
Data augmentation	Albumentations (jitter, blur, noise, compression, flips, affine)

### Comparison with the SOTA methods

To provide a comprehensive and rigorous evaluation, we compare LiteCrackSeg against 11 SOTA methods spanning the principal paradigms: (1) edge-aware segmentation backbones and encoder–decoder CNNs (UNet++ [52], DeepLabV3+ [53]); (2) attention-augmented UNet variant (AttuNet [54]); (3) specialized CNN-based crack segmenters tailored to this domain (RINDNet [55], SFIAN [19]); and (4) contemporary CNN–transformer hybrids that model long-range dependencies (CT-CrackSeg [17], DTrCNet [15], SimCrack [56], CrackSCF [25]). This diverse benchmarks enables a fair assessment against foundational architectures, attention-based enhancements, crack-specific CNN designs, and recent hybrid models, capturing the full spectrum from classic segmentation networks to lightweight transformer-infused paradigms. To further strengthen the comparison with recent transformer-based segmentation architectures, we additionally evaluated two representative models, TransUNet [57] and SegFormer-B0 [58]. The results are integrated into the comparison tables in this section across three benchmark crack datasets (DeepCrack, CrackMap, and TUT), allowing a direct comparison between LiteCrackSeg and modern transformer-based segmentation baselines.

**Fig 6:**
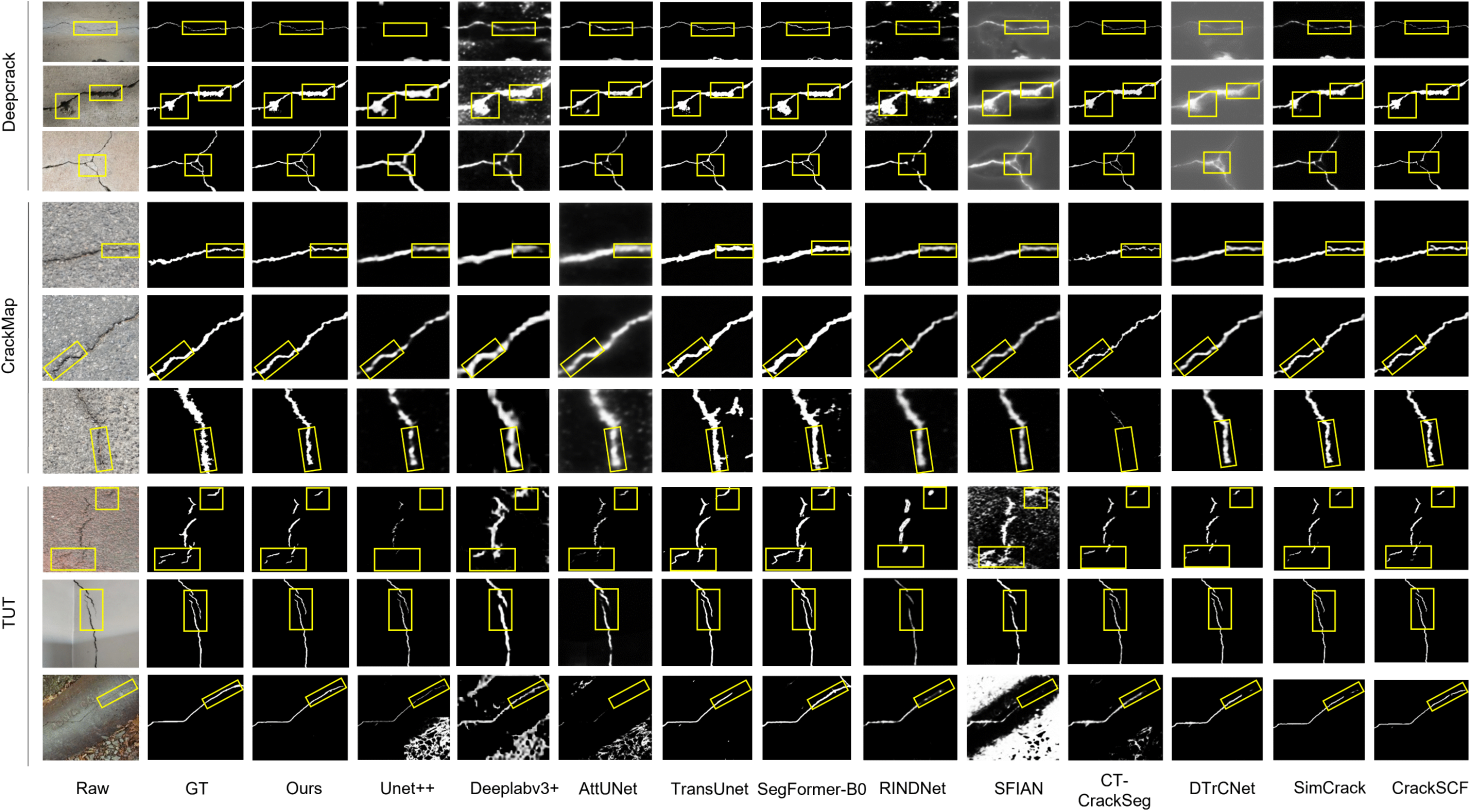
Qualitative results on DeepCrack, CrackMap, and TUT datasets. Yellow boxes highlight key areas.

Qualitative results comparing our proposed model with SOTA methods on the DeepCrack, CrackMap, and TUT datasets are shown in Fig 6. Across all three datasets, our model demonstrates robust performance, accurately segmenting cracks even in challenging conditions with heavy shadows, uneven illumination, and significant background noise. As highlighted by the yellow boxes in Fig 6, the predicted masks from LiteCrackSeg closely match the ground-truth annotations, maintaining better continuity and generating fewer false positives than competing methods.

### How does the proposed model handle diverse crack morphologies in cluttered environments?

To address the challenge of segmenting diverse and low-contrast cracks on resource-constrained devices, we evaluate the proposed model on the DeepCrack dataset, which features a wide variety of crack types under cluttered conditions. The model demonstrates strong performance in handling fine details and complex structures while maintaining computational efficiency. As reported in Table 4, LiteCrackSeg achieves SOTA performance across all metrics on this dataset, surpassing the second-best approach by +0.46% ODS, + 0.57% OIS, + 0.38% Precision, + 0.97% Recall, + 0.73% F1, and +0.37% mIoU. The superior recall gain demonstrates our model’s ability to capture fine and elongated crack details often missed in cluttered scenes. While CrackSCF [25] also performed well on this dataset, our method was better at segmenting detailed cracks, as visualized in Fig 6, where our model produces crisp and continuous boundaries. We attribute these gains to the synergy of the morphology-aware MobileViT (MAM-ViT) bottleneck with dual-branch DSConv, which explicitly models the broad tubular structure of the cracks, while the global context from the MobileViT blocks ensures long-range connectivity is maintained, significantly reducing the fragmentation issues seen in pure CNN-based methods. The multi-scale decoder then effectively refines these strong initial predictions, resulting in superior boundaries. Together, these components allow the network to maintain region-level coherence while retaining edge sharpness, even in challenging areas with low contrast and background clutter.

**Table 4:** Comparison results on DeepCrack dataset. Best results are in bold.

Methods	ODS	OIS	P	R	F1	mIoU
UNet++ [52]	0.8419	0.8893	0.8474	0.8533	0.8503	0.8621
DeepLabV3+ [53]	0.7727	0.8354	0.7363	0.8323	0.7813	0.8565
AttuNet [54]	0.8153	0.8379	0.8066	0.8069	0.8068	0.8578
TransUNet [57]	0.7065	0.7136	0.7246	0.8170	0.7680	0.7961
SegFormer-B0 [58]	0.8055	0.8180	0.8379	0.8597	0.8487	0.8410
RINDNet [55]	0.8087	0.8267	0.7896	0.8920	0.8377	0.8391
SFIAN [19]	0.8616	0.8928	0.8549	0.8692	0.8620	0.8776
CT-CrackSeg [17]	0.8819	0.8904	0.9011	0.8895	0.8952	0.8925
DTrCNet [15]	0.8473	0.8512	0.8905	0.8251	0.8566	0.8661
SimCrack [56]	0.8570	0.8722	0.8984	0.8549	0.8761	0.8744
CrackSCF [25]	0.8914	0.8963	0.9147	0.9013	0.9079	0.9001
Ours	**0.8955**	**0.9014**	**0.9182**	**0.9101**	**0.9141**	**0.9034**

Note: TransUNet was evaluated using cropped inputs of 512 × 512 due to architectural constraints, while all other models were evaluated at the original 544 × 384 resolution.

### How effectively does the proposed model capture thin and elongated crack structures?

The CrackMap dataset presents a different challenge, with its focus on thin and elongated bituminous cracks, which probe a model’s ability to recover fine structures and maintain connectivity along long trajectories. As shown in Table 5, our proposed method surpasses all competing SOTA models, exceeding the second-best by +0.34% ODS, + 0.27% OIS, + 0.55% Precision, + 0.25% Recall, + 0.40% F1, and +0.27% mIoU. While SimCrack [56] and CrackSCF [25] also performed well on this dataset, our method was better at segmenting detailed cracks, as seen in Fig 6. The performance gains on this dataset highlight the strength of the Dynamic Snake Convolutions in the MAM-ViT bottleneck, which are specifically designed to deform their receptive fields to trace fine, curvilinear paths. This explicit geometric modeling allows the network to capture faint crack signals that might be missed by standard convolutions. Furthermore, the ECA modules on the skip connections amplify these faint crack features before they are passed to our attention-guided fusion, which meticulously preserves these high-frequency details during reconstruction, preventing the over-smoothing that often plagues other models on such delicate structures.

**Table 5:** Comparison results on CrackMap dataset. Best results are in bold.

Methods	ODS	OIS	P	R	F1	mIoU
UNet++ [52]	0.7030	0.7222	0.6773	0.7209	0.6984	0.7631
DeepLabV3+ [53]	0.6617	0.6793	0.5853	0.7532	0.6587	0.7460
AttuNet [54]	0.6618	0.6773	0.6233	0.6970	0.6581	0.7454
TransUNet [57]	0.7080	0.7254	0.6844	0.7489	0.7152	0.7622
SegFormer-B0 [58]	0.6868	0.7087	0.6625	0.7124	0.6865	0.7477
RINDNet [55]	0.6745	0.6943	0.6023	0.7586	0.6699	0.7425
SFIAN [19]	0.7200	0.7465	0.6715	0.7668	0.7160	0.7748
CT-CrackSeg [17]	0.7289	0.7373	0.6911	0.7669	0.7270	0.7785
DTrCNet [15]	0.7328	0.7413	0.6912	0.7681	0.7276	0.7812
SimCrack [56]	0.7559	0.7625	0.7380	0.7672	0.7523	0.7963
CrackSCF [25]	0.7595	0.7651	0.7390	0.7708	0.7546	0.7987
Ours	**0.7621**	**0.7672**	**0.7431**	**0.7727**	**0.7576**	**0.8009**

### How well does the proposed model generalize across diverse real-world conditions?

To evaluate LiteCrackSeg’s robustness in addressing morphological challenges under varied real-world conditions, we used the TUT dataset which spans eight scenarios (bitumen, cement, bricks, runway, tiles, metal, blades, and pipes) and contains diverse imaging conditions, making it a rigorous test of generalization. Success on this benchmark requires more than just accuracy on a single domain; it demands a robust architecture that is resilient to diverse textures, lighting, and significant background noise. As reported in Table 6, our method surpasses all competing SOTA models, exceeding the second-best by +0.94% ODS, + 0.91% OIS, + 0.13% Precision, + 1.94% Recall, + 1.03% F1, and +0.74% mIoU. Although CrackSCF [25] remains competitive, the consistent ODS/OIS margins indicate that our method delivers more stable performance and preserves boundary fidelity across diverse scenes as seen in Fig 6.

**Table 6:** Comparison results on TUT dataset. Best results are in bold.

Methods	ODS	OIS	P	R	F1	mIoU
UNet++ [52]	0.7702	0.7948	0.7725	0.7974	0.7848	0.8160
DeepLabV3+ [53]	0.7504	0.7777	0.7155	0.8231	0.7655	0.8177
AttuNet [54]	0.7539	0.7795	0.7558	0.7945	0.7747	0.8204
TransUNet [57]	0.8016	0.8032	0.7964	0.8129	0.8046	0.8340
SegFormer-B0 [58]	0.7932	0.7973	0.7843	0.8096	0.7967	0.8283
RINDNet [55]	0.7531	0.7891	0.7872	0.7665	0.7767	0.8051
SFIAN [19]	0.7290	0.7513	0.7715	0.7367	0.7537	0.7896
CT-CrackSeg [17]	0.7940	0.7996	0.8202	0.8195	0.8199	0.8301
DTrCNet [15]	0.7987	0.8073	0.7972	0.8441	0.8202	0.8331
SimCrack [56]	0.7984	0.8090	0.8051	0.8371	0.8208	0.8334
CrackSCF [25]	0.8202	0.8266	0.8282	0.8484	0.8382	0.8473
Ours	**0.8280**	**0.8342**	**0.8293**	**0.8652**	**0.8469**	**0.8536**

### Statistical analysis

To further assess the consistency of the performance improvements, we compared LiteCrackSeg with representative transformer-based baselines across multiple benchmark datasets (DeepCrack, CrackMap, and TUT). As shown in Tables 4–6, LiteCrackSeg consistently achieves the highest performance across the evaluated metrics, including ODS, OIS, F1-score, and mIoU.

The improvements are observed consistently across datasets with different crack characteristics and imaging conditions, suggesting that the gains are not dataset-specific but reflect a more robust segmentation capability of the proposed architecture.

The statistical analysis of the per-image F1 score distributions (*N* = 282) provides further evidence of this performance gap. While Table 6 presents the aggregate dataset-wide metrics, this granular analysis accounts for the variance across individual scenes. A Wilcoxon signed-rank test reveals that the improvement is highly significant (*p* < 0.001), indicating that the observed gains are not products of random variance but represent a systematic shift in model capability. LiteCrackSeg achieves a mean per-image F1 of 0.8187 with a 95% confidence interval (CI) of [0.808, 0.829], while CrackSCF achieves a mean of 0.8064 (95% CI: [0.795, 0.817]). The separation between these performance distributions, alongside the extremely low *p*-value, confirms the reliability of our model’s lead. This consistent advantage is attributable to the proposed hybrid architecture, where the MAM-ViT bottleneck maintains accuracy across different morphologies and the attention-guided decoder suppresses background noise. Collectively, these results demonstrate that LiteCrackSeg delivers robust and statistically validated performance across diverse real-world inspection scenarios.

**Fig 7:**
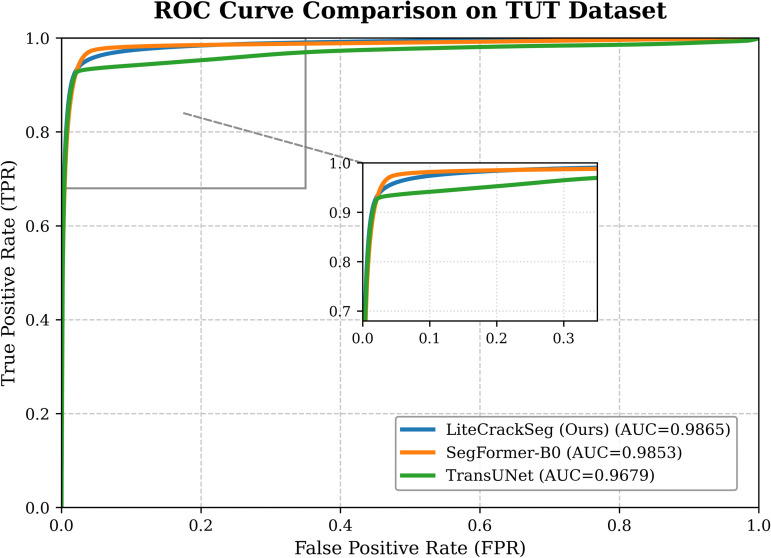
ROC curves for crack segmentation on the TUT dataset comparing LiteCrackSeg, SegFormer-B0, and TransUNet. LiteCrackSeg achieves the highest AUC, demonstrating stronger discriminative capability in distinguishing crack from non-crack pixels.

To further evaluate the discriminative capability of the proposed model, we performed a Receiver Operating Characteristic (ROC) analysis on the TUT dataset. The ROC curves are shown in Fig 7. Although the differences among the methods are relatively small, LiteCrackSeg achieves the highest Area Under the Curve (AUC) of 0.9865, slightly outperforming SegFormer-B0 (0.9853) and clearly surpassing TransUNet (0.9679).

ROC analysis further confirms the strong discriminative power of the proposed architecture. As illustrated in Fig 7, LiteCrackSeg consistently maintains a higher true positive rate across different false positive rates compared with the baseline methods. The steeper ROC curve indicates improved sensitivity in detecting crack pixels while maintaining low false positive rates.

To assess cross-dataset generalization, we trained on TUT dataset and evaluated on the DeepCrack and CrackMap test sets without fine-tuning. As shown in Table 7, LiteCrackSeg consistently outperforms SOTA models across most metrics, demonstrating stronger generalization despite inevitable distribution differences. When evaluated on DeepCrack, LiteCrackSeg achieves an ODS of 0.7983 and F1 of 0.8123, surpassing CrackSCF by +1.69% and +2.77%, respectively. Although a moderate performance drop is observed compared with its in-domain score (0.9141 F1), this is expected due to substantial variations in texture, contrast, and illumination between TUT and DeepCrack. The model maintains high recall (0.9006) but slightly reduced precision (0.7398), indicating strong crack sensitivity with minimal over-segmentation under unseen conditions. On the CrackMap dataset, both models experience a modest decline, but LiteCrackSeg maintains the highest overall consistency, particularly in OIS and F1, reflecting effective adaptation to thin and low-contrast cracks. Overall, the model demonstrates strong generalization capability, relative to each dataset’s in-domain F1, the model retains 89% on DeepCrack and 97% on CrackMap dataset. This high level of performance preservation confirms that the hybrid CNN–transformer design effectively balances adaptability and robustness across different domains.

**Table 7:** Cross-dataset generalization performance comparison (trained on TUT dataset). Best results are in bold.

Test Dataset	Models	ODS	OIS	P	R	F1	mIoU
DeepCrack	CT-CrackSeg [17]	0.7757	0.8041	0.6731	0.8809	0.7631	0.8190
	DTrCNet [15]	0.7971	0.8077	0.7002	**0.9120**	0.7922	0.8305
	SimCrack [56]	0.7887	0.8136	0.7085	0.9077	0.7958	0.8264
	CrackSCF [25]	0.7848	0.8071	0.7030	0.9010	0.7898	0.8239
	Ours	**0.7983**	**0.8224**	**0.7398**	0.9006	**0.8123**	**0.8324**
CrackMap	CT-CrackSeg [17]	0.7157	0.7362	0.6095	0.7565	0.6752	0.7754
	DTrCNet [15]	0.7160	0.7509	0.6284	0.7757	0.6943	0.7700
	SimCrack [56]	0.7136	0.7480	0.6355	0.7692	0.6960	0.7687
	CrackSCF [25]	0.7243	0.7536	0.6453	0.7855	0.7085	0.7753
	Ours	**0.7246**	**0.7578**	**0.6854**	**0.7949**	**0.7361**	**0.7801**

### Model complexity

The complexity analysis of the proposed model against SOTA models is shown in Table 8. We quantify efficiency by reporting parameter counts (millions, M), floating-point operations (GFLOPs), and frames per second (FPS) at an input resolution of 512 × 512. LiteCrackSeg requires only 2.72 M parameters and 3.23 GFLOPs, establishing the lowest computational footprint among all evaluated methods. Compared to the next smallest model SegFormer-B0, our method achieves reductions of roughly 26% and 61% in parameters and GFLOPs, respectively. While SegFormer-B0 achieves a notably high throughput of 189 FPS, our earlier quantitative results demonstrate that it struggles to capture complex crack topologies, falling significantly behind top-performing models like LiteCrackSeg and CrackSCF across key accuracy metrics. In contrast, LiteCrackSeg achieves the highest overall segmentation accuracy while maintaining a robust real-time processing capability of 56 FPS on an NVIDIA GeForce RTX 4090 GPU. Overall, LiteCrackSeg provides an optimal balance between a minimal computational footprint, high segmentation accuracy, and practical real-time throughput for resource-constrained devices.

**Table 8:** Comparison of complexity with other methods. Best results are in bold.

Methods	Parameters	GFLOPs	FPS
UNet++ [52]	9.16M	139.61G	22
DeepLabV3+ [53]	59.39M	88.53G	29
AttuNet [54]	57.16M	541.34G	24
TransUNet [57]	179M	394G	51
SegFormer-B0 [58]	3.71M	8.40G	**189**
RINDNet [55]	59.34M	695.77G	11
SFIAN [19]	13.63M	52.36G	35
CT-CrackSeg [17]	22.88M	39.47G	28
DTrCNet [15]	63.45M	123.20G	47
SimCrack [56]	29.58M	70.66G	29
CrackSCF [25]	4.79M	9.26G	36
Ours	**2.72M**	**3.23G**	56

### Robustness analysis under noisy conditions

To evaluate reliability in real-world scenarios corrupted by sensor interference or environmental factors, we conducted a systematic robustness experiment against SegFormer-B0. We subjected the test dataset to Gaussian noise to simulate dense electronic interference, and Salt-and-Pepper noise to mimic impulsive transmission errors.

**Fig 8:**
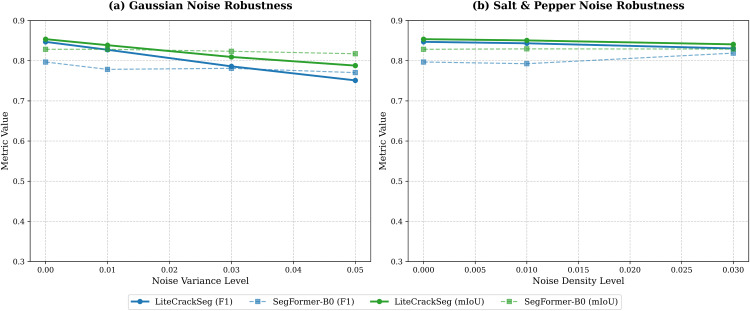
Robustness comparison under varying noise conditions. **(a)** Performance metrics under Gaussian noise ($\sigma^{2}$). **(b)** Performance metrics under Salt-and-Pepper noise **(*d*)**. Solid lines represent LiteCrackSeg (Ours), while dashed lines denote the SegFormer-B0 baseline.

The quantitative results, visualized in Fig 8 and detailed in Table 9, reveal nuanced robustness profiles. Under impulsive Salt-and-Pepper noise (Fig 8(b)), LiteCrackSeg demonstrates superior resilience, maintaining a clear performance gap over SegFormer-B0. At both the 0.01 and 0.03 noise levels, LiteCrackSeg consistently achieves higher ODS, F1, and mIoU scores. This stability stems from the MAM-ViT bottleneck; its Dynamic Snake Convolutions (DSConv) effectively trace crack paths using local morphological continuity, successfully bridging gaps caused by isolated corrupted pixels.

**Table 9:** Quantitative robustness comparison between LiteCrackSeg and SegFormer-B0 under Gaussian and Salt-and-Pepper noise. Best results are in bold.

Noise Type	Level	Method	ODS	OIS	P	R	F1	mIoU
clean	0	SegFormer-B0 [58]	0.7932	0.7973	0.7843	0.8096	0.7967	0.8283
		LiteCrackSeg	**0.8280**	**0.8342**	**0.8293**	**0.8652**	**0.8469**	**0.8536**
Gaussian noise	0.01	SegFormer-B0 [58]	0.7932	0.7967	0.8042	0.7542	0.7784	0.8284
		LiteCrackSeg	**0.8063**	**0.8090**	**0.8490**	**0.8065**	**0.8272**	**0.8386**
	0.03	SegFormer-B0 [58]	**0.7853**	**0.7889**	0.7975	**0.7652**	0.7810	**0.8234**
		LiteCrackSeg	0.7591	0.7612	**0.8459**	0.7338	**0.7859**	0.8093
	0.05	SegFormer-B0 [58]	**0.7753**	**0.7792**	0.7836	**0.7574**	**0.7703**	**0.8173**
		LiteCrackSeg	0.7210	0.7231	**0.8391**	0.6796	0.7510	0.7878
Salt_pepper noise	0.01	SegFormer-B0 [58]	0.7948	0.7986	0.8005	0.7846	0.7925	0.8294
		LiteCrackSeg	**0.8237**	**0.8281**	**0.8393**	**0.8473**	**0.8433**	**0.8505**
	0.03	SegFormer-B0 [58]	0.7940	0.7981	0.8122	**0.8255**	0.8188	0.8290
		LiteCrackSeg	**0.8092**	**0.8121**	**0.8484**	0.8134	**0.8305**	**0.8407**

Conversely, dense Gaussian noise (Fig 8(a)) exposes a vulnerability under extreme degradation. While LiteCrackSeg retains its lead at a lower variance of 0.01, its performance drops more steeply at higher variances. At the 0.03 level, SegFormer-B0 overtakes LiteCrackSeg in ODS (0.7853 vs. 0.7591) and OIS, although our model retains a marginal lead in F1 score (0.7859 vs. 0.7810). However, at the severe 0.05 level, SegFormer-B0 surpasses LiteCrackSeg across most metrics, including ODS (0.7753 vs. 0.7210) and F1 (0.7703 vs. 0.7510). This inversion occurs because DSConv relies heavily on local spatial gradients to deform its receptive field. Dense, high-variance Gaussian noise disrupts these gradients, confusing the morphology-aware sampling trajectories and causing fragmented predictions. In contrast, the global self-attention in pure ViT architectures like SegFormer acts as a spatial low-pass filter, providing inherent mathematical resistance to dense, uniform noise.

Overall, LiteCrackSeg is highly robust to impulsive artifacts, though extremely noisy sensor environments may require preliminary denoising to preserve the local gradients essential for morphology-aware feature extraction.

### Ablation study

To validate key components, we performed a comprehensive ablation study on the TUT dataset. We first analyze the contribution of each major architectural component, then examine the specific configuration of our novel MAM-ViT bottleneck, and finally justify our choice of loss function.

**Fig 9:**
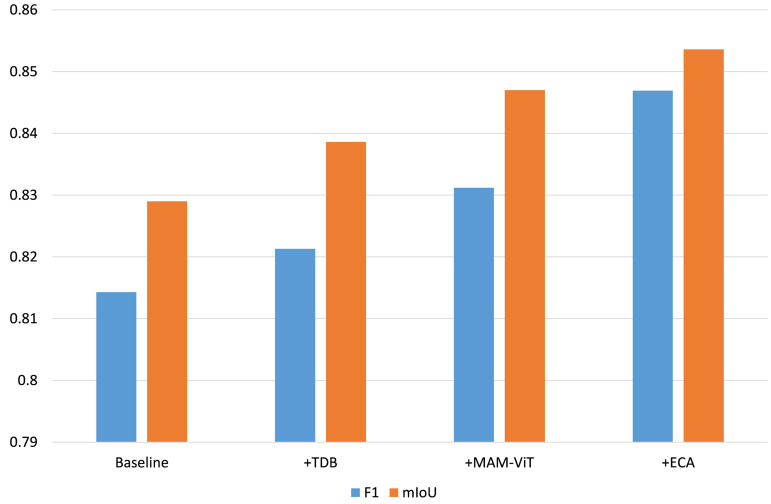
Ablation analysis on TUT dataset: Effects of TDB, MAM-ViT, and ECA on F1 and mIoU.

To evaluate the main architectural components of our model, we begin with a baseline that replaces the MAM-ViT bottleneck by a light MV2/ MobileViT block without DSConv, disables the TDB by using simple CNN refiners in all upsampling blocks, and removes ECA from skip pathways. This conservative configuration establishes a clean reference in which morphology-aware extraction, transformer-like refinement, and channel reweighting are absent, with results shown in Table 10 and visualized in Fig 9.

**Table 10:** Ablation study of main architectural components. Best results are in bold.

Methods	ODS	OIS	P	R	F1	mIoU
Baseline	0.8074	0.8092	0.8125	0.8161	0.8143	0.8290
+ TDB	0.8106	0.8135	0.8194	0.8233	0.8213	0.8386
+ MAM-ViT	0.8274	0.8321	0.8212	0.8414	0.8312	0.8470
+ ECA (Full LiteCrackSeg)	**0.8280**	**0.8342**	**0.8293**	**0.8652**	**0.8469**	**0.8536**

Adding the TDB further improves multi-scale feature quality by injecting local self-attention and a depthwise-enhanced feed-forward path at each decoding stage. Relative to the baseline, we observe gains on TUT of roughly +0.39% ODS, + 0.53% OIS, + 0.84% Precision, + 0.87% Recall, + 0.85% F1, and +1.14% mIoU, indicating that the TDB, through its local self-attention and spatially aware feed-forward layers, refines crack detail during upsampling, sharpening thin boundaries and reducing fragmentation.

Next, enabling MAM-ViT (with DSConv) at the bottleneck yields a consistent uplift across all metrics, reflecting better encoding of curvilinear, tubular structures that dominate crack morphology. We observe gains of +2.03% ODS, + 2.24% OIS, + 0.23% Precision, + 2.15% Recall, + 1.19% F1, and +0.99% mIoU. The dynamic offsets in DSConv allow the network to efficiently handle local curvature while preserving thin segments, improving boundary quality (ODS/OIS).

Finally, introducing ECA on the skip features provides the Full LiteCrackSeg with a channel reweighting before fusion emphasizes informative channels and suppresses nuisance responses, which translates into further gains of around +0.07% ODS, + 0.25% OIS, + 0.98% Precision, + 2.75% Recall, + 1.85% F1, and +0.77% mIoU compared to the TDB variant. The cumulative effect produces steady improvements on all six metrics. In combination, MAM-ViT, TDB, and ECA are complementary: the bottleneck captures global/tubular structure, TDB recovers crisp detail across scales, and ECA focuses fusion on the most discriminative channels.

Building on the importance of the MAM-ViT bottleneck established in Table 10, we isolate its core operator by (i) replacing the dual-branch DSConv with a standard deformable convolution, and (ii) varying the DSConv kernel length K∈{3,5,7}, as shown in Table 11. Using DSConv with (*K* = 5) yields the best overall performance, improving over deformable convolution by +1.05% ODS (0.8280 vs. 0.8193), + 0.89% OIS (0.8342 vs. 0.8268), + 0.69% Precision (0.8293 vs. 0.8236), + 1.55% Recall (0.8652 vs. 0.8518), + 1.04% F1 (0.8469 vs. 0.8375), and +0.71% mIoU (0.8536 vs. 0.8475). The higher Recall and F1 indicate that the dual, orthogonal DSConv branches better trace thin, elongated crack centerlines and reduce fragmentation compared to a single deformable operator.

**Table 11:** Ablation of the MAM-ViT bottleneck: DSConv vs. deformable convolution and DSConv kernel length *K*. Best results are in bold.

Methods	ODS	OIS	P	R	F1	mIoU
Deformable convolution	0.8193	0.8268	0.8236	0.8518	0.8375	0.8475
DSConv (*K* = 3)	0.8246	0.8291	**0.8387**	0.8465	0.8426	0.8509
DSConv (*K* = 5)	**0.8280**	**0.8342**	0.8293	**0.8652**	**0.8469**	**0.8536**
DSConv (*K* = 7)	0.8276	0.8336	0.8341	0.8573	0.8455	0.8529

Among DSConv variants, (*K* = 3) attains the highest Precision (0.8387) but sacrifices Recall (0.8465), yielding a lower F1 (0.8426). Increasing to (*K* = 7) enlarges the receptive path and comes close to the best F1 (0.8455 vs. 0.8469 for (*K* = 5), but slightly reduces ODS/OIS/mIoU. Overall, (*K* = 5) provides the best balance for crack morphology.

To optimize for the severe class imbalance in crack segmentation, we evaluated several loss functions, with the results presented in Table 12. While standard BCE, Dice, and Focal losses provide strong baselines, the Tversky loss offers direct control over the precision-recall trade-off. Based on our analysis, the Tversky loss (α=0.75) was selected due to its superior balance between precision and recall. Compared with the Tversky loss (α=0.85), the Precision metric is 4.46% lower, but the F1 metric increased by 1.41%. Therefore, the α=0.75 setting was used during training, as the improved F1-score indicates a more robust and superior segmentation performance.

**Table 12:** Loss-function ablation on the TUT dataset for LiteCrackSeg. Best results are in bold.

Methods	ODS	OIS	P	R	F1	mIoU
BCE Loss	0.8268	**0.8382**	0.8291	0.8572	0.8429	0.8528
Dice Loss	0.8246	0.8337	0.8258	0.8637	0.8443	0.8512
Focal Loss	0.8215	0.8362	0.8252	0.8595	0.8420	0.8495
Tversky Loss (α=0.65)	0.8237	0.8319	0.7980	**0.8764**	0.8354	0.8505
Tversky Loss (α=0.75)	**0.8280**	0.8342	0.8293	0.8652	**0.8469**	**0.8536**
Tversky Loss (α=0.85)	0.8172	0.8194	**0.8624**	0.8092	0.8350	0.8456

## Limitations and discussions

Our extensive experiments demonstrate that LiteCrackSeg achieves a SOTA balance of accuracy and efficiency, leveraging its hybrid architecture and the novel MAM-ViT bottleneck for robust segmentation. The lightweight design and high FPS make it a strong candidate for real-world deployment on resource-constrained devices. However, we have identified several limitations that pave the way for future research.

**Fig 10:**
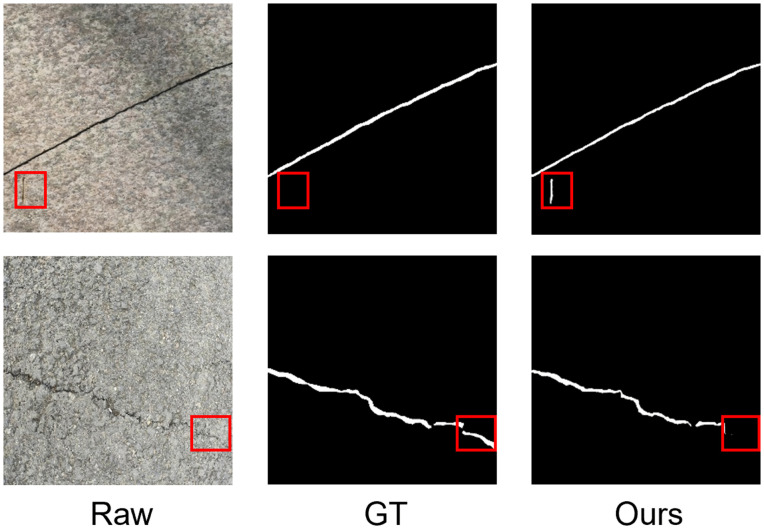
Examples of LiteCrackSeg misdetections (FP) and misses (FN). The critical regions are marked with red boxes.

First, the model can struggle with distinguishing between true cracks and crack-like artifacts such as surface scratches in pavement, as shown in the first row of Fig 10 (highlighted in red). These features share geometric properties with cracks, occasionally leading to false positives. Future work could address this by incorporating a contrastive learning objective, which would train the model to learn a more discriminative feature space that better separates true defects from visually similar but structurally distinct background noise.

Second, the model has a detection threshold for extremely fine or low-contrast cracks. As seen in the second row of Fig 10 (highlighted in red), the model sometimes fails to capture the full extent of a crack where it becomes faint and blends into the background texture. This results in an incomplete segmentation, suggesting that while the MAM-ViT DSConv component is effective, its signal can be lost when crack features fall below a certain intensity. A potential research direction is to explore topological continuity losses that would explicitly penalize fragmented predictions and encourage the model to complete faint paths between more confident crack segments.

In addition to these model-specific challenges, several broader limitations should be acknowledged. The proposed LiteCrackSeg framework relies on supervised learning and therefore requires high-quality pixel-level annotations for effective training. Producing such detailed annotations for large-scale infrastructure datasets can be labor-intensive. Furthermore, although the proposed architecture is designed to be lightweight, deployment on extremely constrained edge devices may still require additional optimization techniques such as pruning or quantization. Finally, while the robustness experiments demonstrate resilience to synthetic noise, further evaluation under additional real-world degradations (e.g., motion blur, severe illumination changes, or weather-related artifacts) would provide a more comprehensive assessment of the model’s practical reliability. Addressing these aspects represents an important direction for future work.

## Conclusion

In this paper, we propose LiteCrackSeg for efficient pixel-level crack segmentation. The proposed model features an encoder-decoder framework, which integrates a lightweight hybrid MobileViT encoder for multi-scale local–global representation learning. A key component of our design is the Morphology-Aware MobileViT (MAM-ViT) bottleneck, which utilizes dual-branch Dynamic Snake Convolutions (DSConv) to explicitly capture the slender, tubular geometry characteristic of cracks. To address class imbalance and improve discrimination between crack and background pixels, we integrated a Tversky loss function. This architecture was validated through extensive experiments on three public benchmark datasets, where it demonstrated robust segmentation capabilities while maintaining a minimal computational footprint. Despite its strong performance, future work could explore its application under more adverse conditions and focus on deployment and validation on mobile and edge devices for real-world inspection. Furthermore, the core principles of the hybrid MobileViT encoder and the MAM-ViT bottleneck could be adapted to other challenging segmentation tasks involving fine, curvilinear structures, such as retinal vessel segmentation in medical imaging.

Despite the strong performance achieved by LiteCrackSeg, several limitations remain. In particular, the model can occasionally confuse crack-like artifacts such as scratches with true cracks, and extremely faint or low-contrast crack segments may not always be fully recovered. Additionally, the proposed framework relies on supervised learning with pixel-level annotations, which can be costly to obtain for large infrastructure datasets. Future work will investigate strategies such as contrastive learning to better distinguish cracks from similar artifacts, topological continuity constraints to improve detection of faint crack segments, and lightweight model compression techniques to further enhance deployment on resource-constrained edge devices. These directions could further improve robustness and practical applicability of crack segmentation systems.
